# High CD74 expression correlates with ZAP70 expression in B cell chronic lymphocytic leukemia patients

**DOI:** 10.1007/s12032-013-0560-5

**Published:** 2013-04-10

**Authors:** Aleksandra Butrym, Miroslaw Majewski, Justyna Dzietczenia, Kazimierz Kuliczkowski, Grzegorz Mazur

**Affiliations:** 1 Department of Hematology, Blood Neoplasms and Bone Marrow Transplantation, Wroclaw Medical University, Pasteur 4 Str, 50-367, Wroclaw, Poland; 2 Department of Physiology, Wroclaw Medical University, Wroclaw, Poland; 3 Department of Hematology, Nicolaus Copernicus Hospital, Torun, Poland; 4 Department of Internal, Occupational Diseases and Hypertension, Wroclaw Medical University, Wroclaw, Poland

**Keywords:** CD74, Chronic lymphocytic leukemia, Expression, ZAP70

## Abstract

Chronic lymphocytic leukemia (CLL) is the most common leukemia in adults in Western countries. It is characterized by heterogeneous clinical course of the disease and new prognostic factors are still needed. CD74 plays an important role in signal transduction in B cell proliferation and survival pathway. CD74 expression has been shown in solid tumors and has been connected with poor prognosis and tumor progression. The aim of the study was to evaluate the expression of CD74 in chronic lymphocytic leukemia patients with combination with other known prognostic factors. Expression of CD74 was determined in 90 patients and 28 healthy controls. CD74 expression was significantly higher in CLL group than in controls. There was positive correlation between CD74 and ZAP70 expression (*p* = 0.008). High expression of CD74 was positively correlated with more advanced stage of the disease (*p* = 0.02). No correlation was shown between CD74 and sex, mutational status IgVH and time to first treatment.

## Introduction

Chronic lymphocytic leukemia (CLL) is the most common leukemia in adults in Western countries [1, 2], with very heterogeneous clinical course of CLL from indolent to aggressive [3]. Prognostic factors used currently in practice, such as mutational status of immunoglobulin heavy chain coding genes (IgVH), and expression of CD38 or ZAP70 (zeta-associated protein), have shown their significance in CLL, but are time-consuming and require standardization of laboratory protocol [4, 5]. New prognostic factors are still needed.

CD74, also known as invariant chain, is a transmembrane, nonpolymorphic type II protein, functional in many immunological processes. The most important role of CD74 is regulation of the movement of major histocompatibility complex class II antigens in antigen-presenting cells [6]. CD74 is directly involved in the maturation of B cells through the nuclear factor NF-kappaB [7]. CD74 serves as a high-affinity receptor for the proinflammatory cytokine MIF (macrophage migration inhibitory factor) [8, 9]. Overexpression of MIF occurs in many solid tumors and is associated with tumor progression [10].

CD74 expression occurs in normal tissues on HLA (human leukocyte antigens) II positive cells, including B lymphocytes, monocytes, macrophages, Langerhans’ cells, dendritic cells, activated T cells, and thymic epithelium. CD74 expression was also observed in many cancers, including hematological malignancies (multiple myeloma) [11], as well as solid tumors, such as gastric cancer [12], kidney [13], small cell lung cancer [14], epithelial carcinoma of the thyroid [15], and sarcomas [16]. In all cases, CD74 expression was reported as a prognostic factor reflecting tumor progression and poor clinical prognosis. The aim of this study was to assess the expression of CD74 in chronic lymphocytic leukemia cells in relation to known prognostic factors.

## Materials and methods

The study included 90 patients (44 women and 46 men) with B cell chronic lymphocytic leukemia aged 42–88 years (median 67 years), observed in the Department of Hematology, Blood Neoplasms and Bone Marrow Transplantation Wroclaw Medical University. CLL was diagnosed according to National Cancer Institute Working Group (NCIWG) guidelines [17]. Study was carried out at the diagnosis. Patient had not been treated before. The control group consisted of 28 healthy subjects (11 men and 17 women) aged from 36 to 79 years (median 65 years). Research was carried out in compliance with the Helsinki Declaration. For the study, approval of Bioethical Committee of Wroclaw Medical University, Poland (Approval 521/2008) was obtained. Written informed consent for the study was signed by all the participants. For each patient, the following parameters were analyzed: CD38 expression, IgVH mutational status, ZAP-70, expression, disease stage according to modified Rai criteria, survival time and lymphocyte doubling time (assuming the time grouping value of 6 months), and laboratory parameters [17].

### Flow cytometry

Mononuclear cells from peripheral blood were isolated by gradient separation using Gradisol L. The antibodies used included the following: anti-CD38, anti-CD5, anti-CD23, IgG_1_ (Beckman Coulter), anti-CD74 (Southern Biotech) conjugated with fluorescein isothiocyanate [FITC] (Beckman Coulter), anti-CD19, and IgG_1_ conjugated with phycoerythrin [PE] (DakoCytomation, Germany); mouse isotypic controls were as follows: IgG_2b_, IgG_1_ conjugated with PE (DakoCytomation, Germany) and IgG_1_ conjugated with FITC (Southern Biotech). Expression of antigens was analyzed for fluorescence using a Particle Analyzing System (PAS) flow cytometer (Partec, Germany). The positive expression of CD38 in the test cell population was the presence of the antigen on at least 10 % of the cells [18].

### ZAP70 expression

Cells were incubated for 20 min in the dark with 5 ml of anti-CD19 (IgG_1_ conjugated with phycoerythrin [PE], DakoCytomation, Germany), and then fixed with a solution containing paraformaldehyde (reagent 1, kit IntraPrepTM), shaken and incubated for 15 min at room temperature. Cells were washed and then centrifuged. Supernatant was discarded and then added to the permeabilization reagent B (IntraPrepTM set), leaving 5 min without stirring and then incubated for 15 min at room temperature in the presence of 7.5 ml antibodies anti-ZAP70 (clon SB70, IgG_2b_ conjugated with FITC, Beckman Coulter) or relevant control antibody, isotype-compatible. Cells were again washed. The expression of ZAP70 in the cytoplasm of B cells (CD19^+^) was analyzed by flow cytometry. ZAP70 expression in the cytoplasm of B cells (CD19^+^) was described as positive expression when the presence of ZAP70 expression was detected on at least 30 % of the cells [19].

### Analysis of mutational status of IgVH

The analysis of mutational status was carried out in accordance with European Research Initiative on CLL (ERIC) guidelines for analyzing IgVH in chronic lymphocytic leukemia, published in 2007 in the Leukemia [20]. Taking the cutoff point of 98 % sequence homology test for germline sequences contained in databases, patients were divided into two groups: with mutated IgVH genes (*M* < 98 % homology) and unmutated IgVH genes (*U* > 98 % homology).

### Statistical analysis

The results obtained were subjected to statistical development. For all tested parameters, the average values (*x*), median (*M*), and standard deviations (SD) constant were calculated. Verification of the hypothesis of equality of average individual samples was performed by ANOVA or analysis of variance for groups with heterogeneous variance nonparametric rank sum test, Kruskal–Wallis (homogeneity of variance was checked using Bartlett test). For discrete parameters, the frequency characteristics of the test groups were analyzed with the amendment χ_*df*_^2^ Yate’s appropriate number of degrees of freedom *df* (*df* = (*m* − 1) * (*n* − 1), where *m* number of rows and *n* number of columns. For the analyzed pairs of parameters, Pearson’s or Spearman’s correlation coefficient was calculated. Multivariate analysis was performed using logistic regression (quasi-Newton estimation method). The survival curves were plotted using the Kaplan–Meier method and compared using the log-rank test. The *p* value *p* ≤ 0.05 was considered statistically significant. Statistical analysis was performed using computerized statistical software package EPIINFO ver. 3.4.3 (from 08 Nov 2007).

## Results

Patient and control group characteristic are presented in Table 1.

**Table 1:** Characteristic of patients and control group

Chronic lymphocytic leukemia patients			Total No of pts
Women/men	44/46		90
Age (years)	Range 42–88	Median 67	
Stage Rai	0	17	90
	I	16	
	II	18	
	III	15	
	IV	24	
Hemoglobin (g/dl)	<12.0	25	90
	≥12.0	65	
Platelets × 10^3^/μL	<100	24	90
	≥100	66	
Lactate dehydrogenase	≤1× norm	44	82
	>1× norm	38	
β_2_-microglobulin (mg/l)	≤1× norm	7	76
	>1× norm	69	
C-reactive protein (mg/l)	≤1× norm	50	79
	>1× norm	29	
ZAP70 expression	<30 %	38	86
	≥30 %	48	
CD38 expression	<10 %	71	86
	≥10 %	15	
IgVH mutational status	Unmutated	43	90
	Mutated	44	
	Undetermined	3	
Control group			
Women/men	17/11		28
Age (years)	Range 36–79	Median 65	

### CD74 expression

**Fig. 1:**
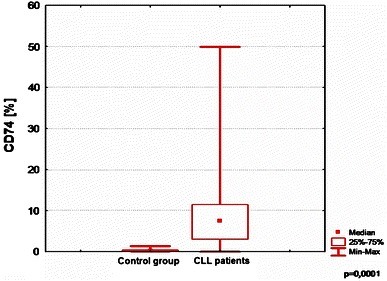
Comparison of CD74 expression between CLL patients and control group

CD74 expression was measured in 90 patients, and it was significantly higher in CLL group comparing to controls (*p* = 0.0001) (Fig. 1; Table 2).

**Table 2:** Comparison between CD74 expression in CLL and control group

	Control group			CLL			*p*
	*M*	Range	*n*	*M*	Range	*n*	
CD74	0.240	0.4–1.39	28	7.48	0.7–49.85	90	0.0001

*M* median value, *n* number of patients

Expression of CD74 was higher in patient with more advanced stage of disease according to Rai classification comparing to group with lower stage of the disease (stages 0 + I + II: mean value 7.73, median 6.66 % vs. stages III + IV: mean value 11.2 %, median 7.9 %; *p* = 0.02).

**Fig. 2:**
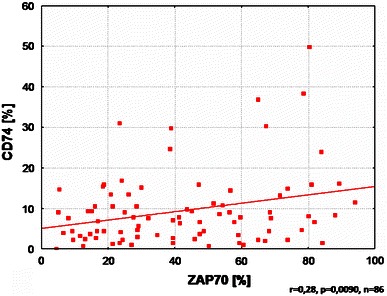
Correlation between CD74 and ZAP70 expression

We also showed positive correlation between CD74 expression and ZAP70 (*p* = 0.008). Percentage of ZAP70-positive CLL cells was also positively correlated with CD74 expression (*r* = 0.28, *p* = 0.009) (Fig. 2).

**Fig. 3:**
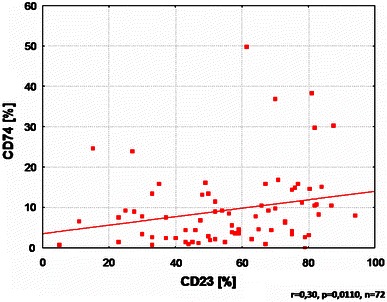
Correlation between CD74 expression and CD23

**Fig. 4:**
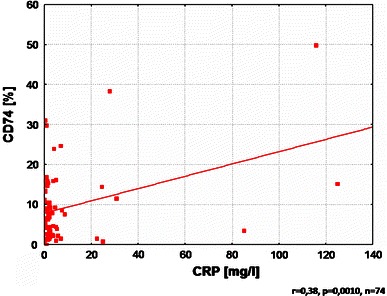
Correlation between CD74 expression and C-reactive protein

Additionally, the level of CD74 expression was positively correlated with CD23 and C-reactive protein level (Figs. 3, 4).

We did not show any relationship between CD74 and age, gender, leukocytosis, hemoglobin level, LDH, IgVH mutational status, CD38 expression, and β2-mikroglobulin (*p* = NS, nonsignificant). There was also no correlation between CD74 expression and patient’s survival (*p* = NS). In multivariate analysis of risk factors affecting survival of CLL patients, CD74 expression did not demonstrate the independent importance associated with patient survival.

## Discussion

CD74 is an integral membrane protein acting as a signaling molecule adjuvant [21, 22] and is involved in the mechanism of tumor cell survival [23–31]. Cytoplasmatic domain of CD74 induces maturation of B cells through activation of transcription mediated by the homodimer of NF-kappaB p65/RelA and its coactivator TAFII105 [7]. Stimulation of CD74 with anti-CD74 or MIF leads to the activation of NF-kappaB, allowing the entry of stimulated B cells in S phase of the cell cycle, increased DNA synthesis, cell division, and increased expression of antiapoptotic proteins of Bcl-2 family [23, 27, 28]. Overexpression of CD74 was reported in various cancers (stomach, kidney, lung and multiple myeloma), suggesting that it can serve as a prognostic factor, with higher CD74 values indicating tumor progression [10, 12–16, 32].

In our study, we observed significantly higher expression of CD74 in comparison with the control group, which is consistent with the results of Binsky et al. [24]. Authors observed increased expression of CD74 measured by flow cytometry on the surface of CLL cells compared to normal B lymphocytes [24]. These results were homogeneous in the whole group of patients, regardless of the clinical parameters, such as stage of disease according to Rai and Binet, the absolute count of lymphocytes, lymphocyte doubling time, prior chemotherapeutic treatment, age, gender, cell morphology, immunoglobulin levels, autoimmunity, and the expression of ZAP70 and CD38 [24]. In the present study, CD74 expression was lower than in the study by Binsky, but this fact could be linked to different antibodies used by authors. Binsky et al. presented results of CD74 expression based on the examples of only few patients, which could also influence the difference in CD74 expression. In present study, we also did not observe the relationship between CD74 expression and the majority of parameters. In contrast, however, we showed a positive correlation between CD74 and stage of the disease as well as the level of C-reactive protein (CRP). As it is known, inflammation is one of the factors that can sustain the proliferation of cancer cells through activation of stromal cells producing growth factors. High expression of CD74 occurs in inflammatory diseases, as well as in tumors associated with inflammation [collected in 33]. MIF binding to CD74 induces NF-kappaB and the subsequent cellular response in the form of secretion of proinflammatory cytokines such as IL-1, IL-6, and TNF (tumor necrosis factor). Increased expression of CD74 correlated with the level of CRP may indicate the important role of inflammation in the CLL.

In this study, we demonstrated significantly higher expression of CD74 in the group of CLL patients as compared to the controls and the expression was positively correlated with ZAP70. Gore et al. [28] have shown that in B cells, the signal cascade initiated by binding of MIF to CD74-CD44 complex causes activation of Syk kinase pathway. Syk kinases belong to the family of tyrosine kinases Syk/ZAP70 that play a key role in the development of B cells. Expression of Syk in nonhematopoietic cells is significant in the pathogenesis of malignant tumors. It was shown that Syk is essential for the activation of the Act-kinase, in a manner dependent on the effector phosphatidylinosytol-3 kinase [34]. The activation of Akt promotes cellular response associated with cell division, suppression of apoptosis, inactivation of cell cycle inhibitors, cyclin induction, and cytokine gene expression [35]. On the molecular level, expression of activated Akt kinase in T cells correlates with increased NF-kappaB function, including increased expression of Bcl-XL [28]. CD74-positive correlation between the expression of CD74 and disease stage as well as ZAP70 indicates the possibility to classify CD74 expression as a novel prognostic factor. It is known that the expression of ZAP70 remains difficult to implement and requires a standardized test procedure.

Binsky et al. [24] showed not only higher expression of CD74 in patients with CLL, regardless of clinical status, but also noted that the expression has led to significant prolongation of leukemic cells survival, which was associated with higher expression of MIF. Stimulation of CD74 by its MIF ligand induces signaling cascade leading to transcription and secretion of interleukin-8, known for its angiogenic and procancerogenic properties [36]. IL-8 through autocrine/paracrine action secondarily increases the survival of leukemic cells. Increased accumulation of CLL cells in the bone marrow, resulting in progressive cytopenia along with the progression of the disease, suggests that underlying mechanism of CLL is also linked with aberrant migration and colonization of leukemic cells to normal hematopoietic niches.

## Conclusions

We showed higher expression of CD74 in CLL and its correlation with known prognostic factor ZAP70 and stage of the disease in chronic lymphocytic leukemia. CD74 can give additional prognostic information on CLL patients apart from ZAP70 and/or IGHV mutational status. It requires further analysis on bigger group, but seems to be useful in clinical practice, as it is quite simple to determine its expression by flow cytometry. As monoclonal antibody against CD74 is being currently used in clinical trials, this molecule can have very important role not only in prognosis, but also in the treatment of CLL in the future.
